# Cloroquina y sus derivados en el manejo de la COVID-19: una revisión sistemática exploratoria

**DOI:** 10.7705/biomedica.5478

**Published:** 2020-12-11

**Authors:** Juan Pimentel, Neil Andersson

**Affiliations:** 1 Community Information and Epidemiological Technologies - Participatory Research at McGill, CIET-PRAM, Department of Family Medicine, McGill University, Montreal, Canada McGill University Department of Family Medicine McGill University Montreal Canada; 2 Facultad de Medicina, Universidad de La Sabana, Chía, Colombia Universidad de la Sabana Facultad de Medicina Universidad de La Sabana Chía Colombia; 3 Escuela de Medicina y Ciencias de la Salud, Universidad del Rosario, Bogotá, D.C., Colombia Universidad del Rosario Escuela de Medicina y Ciencias de la Salud Universidad del Rosario BogotáD.C Colombia; 4 Centro de Investigación de Enfermedades Tropicales, Universidad Autónoma de Guerrero, Acapulco, México Universidad Autónoma de Guerrero Universidad Autónoma de Guerrero Acapulco Mexico

**Keywords:** cloroquina, hidroxicloroquina, coronavirus, revisión sistemática, ensayo clínico, pandemias, SARS-CoV-2, Chloroquine, hydroxychloroquine, coronavirus, systematic review, clinical trial, pandemics, SARS-CoV-2

## Abstract

**Introducción.:**

Recientemente, investigadores chinos y franceses reportaron la eficacia de la cloroquina y la hidroxicloroquina para inhibir la replicación *in vitro* del virus SARS-CoV-2. La diseminación oportuna de la información científica es clave en tiempos de pandemia. Es urgente contar con una revisión sistemática sobre el efecto y la seguridad de estos medicamentos en la COVID-19.

**Objetivo.:**

Describir el estado actual de la literatura científica publicada hasta el 25 de marzo de 2020 sobre el uso de la cloroquina o sus derivados en el manejo de pacientes con COVID-19.

**Materiales y métodos.:**

Se hizo una revisión sistemática exploratoria en PubMed, Embase, Lilacs y 15 bases de datos de la Plataforma de Registros Internacionales de Ensayos Clínicos de la Organización Mundial de la Salud (OMS). Se incluyeron publicaciones empíricas y teóricas en inglés, español, italiano, francés o portugués, y se hizo una síntesis narrativa de los resultados.

**Resultados.:**

Se incluyeron 19 documentos y 24 registros de ensayos clínicos (n=43) de 18.059 pacientes. El 66 % (16/24) de los ensayos están registrados en China. Nueve ensayos evalúan la cloroquina exclusivamente y ocho, la hidroxicloroquina. Los documentos son comentarios (n=9), estudios *in vitro* (n=3), revisiones narrativas (n=2), guías de práctica clínica (n=2), así como una revisión sistemática, un consenso de expertos y un ensayo controlado.

**Conclusiones.:**

Un ensayo clínico pequeño (n=26), no aleatorizado y defectuoso, respalda el uso de la hidroxicloroquina en pacientes con COVID-19. Se requiere de manera urgente tener acceso a los resultados de otros ensayos clínicos para determinar la efectividad y la seguridad de la cloroquina y sus derivados en pacientes con COVID-19.

## Abstract (en)

**Introduction:**

Recently, researchers from China and France reported on the effectiveness of chloroquine and hydroxychloroquine for the inhibition of SARS-CoV-2 viral replication *in vitro.* Timely dissemination of scientific information is key in times of pandemic. A systematic review of the effect and safety of these drugs on COVID-19 is urgently needed.

**Objective:**

To map published studies until March 25, 2020, on the use of chloroquine and its derivates in patients with COVID-19.

**Materials and methods:**

We searched on PubMed, Embase, Lilacs, and 15 registries from the World Health Organization's International Clinical Trials Registry Platform for theoretical and empirical research in English, Spanish, Italian, French, or Portuguese until March 25, 2020, and made a narrative synthesis of the results.

**Results:**

We included 19 records and 24 trial registries (n=43) including 18,059 patients. China registered 66% (16/24) of the trials. Nine trials evaluate chloroquine exclusively and eight hydroxychloroquine. The records are comments (n=9), *in vitro* studies (n=3), narrative reviews (n=2), clinical guidelines (n=2), as well as a systematic review, an expert consensus, and a clinical trial.

**Conclusions:**

One small (n=26), non-randomized, and flawed clinical trial supports hydroxychloroquine use in patients with COVID-19. There is an urgent need for more clinical trial results to determine the effect and safety of chloroquine and hydroxychloroquine on COVID-19.

El 12 de marzo de 2020, la Organización Mundial de la Salud (OMS) declaró como pandemia la enfermedad emergente por coronavirus (COVID-19), causada por el virus SARS-CoV-2 ^1^. El 8 de abril de 2020, las cifras de la pandemia de COVID-19 incluían 1'356.780 casos confirmados, 79.385 muertes y 212 países, áreas o territorios afectados ^2^.

Los efectos antivirales de la cloroquina fueron descritos en la década de 1960, aunque el interés por ellos se ha renovado desde el 2006 ^3^. En la epidemia de SARS del 2003 ^4^, varios investigadores exploraron la efectividad de diferentes moléculas contra el coronavirus SARS-CoV. En el 2004, una concentración efectiva (EC_50_) de 8 µM de cloroquina, antes o después de la exposición de las células al virus, demostró eficacia *in vitro*^5^. Según algunos autores, estos hallazgos terminaron siendo olvidados por razones que no están claras ^6^^,^^7^.

La cloroquina es un inhibidor potente de la mayoría de coronavirus, incluido el SARS-CoV ^8^ y el MERS-CoV ^9^. Se ha demostrado que el medicamento alcaliniza los endosomas celulares, lo cual entorpece algunos pasos de la replicación viral que requieren un pH bajo, como la fusión virus-endosoma y el despojo de la cápside vírica ^10^. Algunos autores han propuesto que la cloroquina también interfiere con la glucosilación de los receptores celulares, como el receptor de la enzima convertidora de angiotensina 2 del SARS-CoV. Además, la cloroquina tiene un efecto inmunomodulador favorable para la eliminación viral ^11^, al reducir la producción de citocinas proinflamatorias y activar los linfocitos T CD8+ anti-SARS-CoV-2 ^12^.

Tanto la cloroquina como la hidroxicloroquina podrían ayudar a controlar la 'tormenta citoquímica' experimentada por los pacientes con COVID-19 ^13^. Dado que la administración de corticoides puede ser deletérea en pacientes con COVID-19 críticamente enfermos ^14^, los efectos inmunomoduladores de la cloroquina y de la hidroxicloroquina representan un valor agregado de estos medicamentos.

El 4 de febrero de 2020, Wang, *et al.,* publicaron un artículo en la revista *Cell Research* que recibió la atención del mundo científico ^11^. Los investigadores evaluaron la actividad de la cloroquina frente al nuevo virus SARS-CoV-2, y encontraron que dosis de 1,13 µM (CE_50_) y de 6,90 µM (CE_90_) del medicamento prevenían la replicación *in vitro* del virus en células Vero E6. Según los autores, tales concentraciones pueden alcanzarse fácilmente en seres humanos con la dosis estándar debido a la penetración favorable del fármaco en los tejidos, incluido el pulmón. Como resultado, la cloroquina es hoy la molécula más usada a nivel internacional para el tratamiento de la COVID-19 ^12^. El 6 de abril de 2020, la cloroquina y la hidroxicloroquina fueron autorizadas por el Ministerio de Salud y Protección Social de Colombia para el tratamiento de esta enfermedad ^15^.

En un comentario publicado el 5 de marzo de 2020, Touret, *et al.,* reportaron 16 registros de ensayos clínicos que exploraban la efectividad y la seguridad de la cloroquina o la hidroxicloroquina en pacientes con COVID-19 en China ^16^. Sin embargo, no se sabe si los resultados de alguno de estos estudios ya están publicados, y tampoco se conocen la efectividad y la seguridad del uso de la cloroquina y de sus derivados en pacientes con COVID-19.

La diseminación oportuna de la información científica es clave en tiempos de pandemia. Es necesaria para orientar a los epidemiólogos, a los profesionales de la salud que atienden a los pacientes infectados, a los investigadores que exploran las tendencias de la enfermedad y los efectos de las intervenciones en salud e, incluso, para reducir el pánico público ^17^. En este sentido, es urgente contar con una revisión sistemática que explore la información científica sobre la efectividad y la seguridad de la cloroquina y sus derivados.

La presente revisión sistemática exploratoria aspira a comunicar al público hispano la información científica disponible hasta el 25 de marzo de 2020 sobre el papel de la cloroquina y sus derivados en el manejo de la COVID-19.

## Materiales y métodos

En la revisión se siguieron los pasos propuestos por Arksey, *et al.*^18^ y Levac ^19^:

- determinar la pregunta de investigación,
- buscar los documentos relevantes,
- seleccionar los estudios,
- extraer los datos, y
- resumir y reportar los resultados.

La revisión respondió a la pregunta: ¿cuál es el estado actual de la literatura científica sobre el uso de la cloroquina o sus derivados en el manejo de pacientes con COVID-19? Para responderla, previamente se desarrolló un protocolo (disponible por pedido a los autores).

### Criterios de inclusión y exclusión

Los criterios de inclusión fueron:

- publicaciones con datos empíricos *(in vitro* o *in vivo)* o publicaciones teóricas (revisiones narrativas, comentarios, cartas al editor);
- que la publicación estudiara o discutiera el papel de la cloroquina o de cualquiera de sus derivados (hidroxicloroquina, amodiaquina, imiquimod o primaquina);
- que presentara resultados de investigación sobre el COVID-19 o el SARS-CoV-2, y
- que estuviera en inglés, español, italiano, francés o portugués.

También se incluyeron otros idiomas cuando el resumen estaba en cualquiera de los idiomas considerados en la revisión.

No se consideró límite de fecha en la búsqueda y la última actualización correspondió al 25 de marzo de 2020.

### Estrategia de búsqueda

La búsqueda incluyó las bases de datos PubMed, Embase a través de Ovid, y Lilacs. Usamos operadores booleanos y palabras clave de acuerdo con cada sistema de datos. El algoritmo de búsqueda está disponible en el archivo suplementario 1. Además, se incluyeron las referencias citadas en los documentos si cumplían con los criterios de inclusión y no se habían detectado previamente, así como dos referencias adicionales publicadas después del 25 de marzo sugeridas por expertos en el tema.

Se buscaron los registros de estudios en 15 bases de datos de la Plataforma de Registros Internacionales de Ensayos Clínicos de la OMS ^20^: Chinese Clinical Trial Registry, U.S. National Library of Medicine, International Standard Randomised Controlled Trial Number (ISRCTN), EU Clinical Trials Register (EU-CTR), Japan Primary Registries Network (JPRN), Australian New Zealand Clinical Trials Registry (ANZCTR), Brazilian Clinical Trials Registry (ReBec), Clinical Research Information Service (CRiS), Republic of Korea, Clinical Trials Registry, India (CTRI), Cuban Public Registry of Clinical Trials (RPCEC), German Clinical Trials Register (DRKS), Iranian Registry of Clinical Trials (IRCT), The Netherlands National Trial Register (NTR), Pan African Clinical Trial Registry (PACTR), y Peruvian Clinical Trial Registry (REPEC). Estos registros cumplen con los criterios específicos de contenido, calidad y validez, accesibilidad y capacidad técnica y administrativa ^20^.

### Selección de estudios y extracción de datos

Usando la aplicación web de acceso gratuito para la gestión de revisiones sistemáticas Rayyan ^21^, se revisaron y escogieron independientemente los títulos y los resúmenes de las publicaciones candidatas. Los investigadores resolvieron discrepancias mediante discusión y consenso. Posteriormente, se eliminaron los duplicados y se obtuvieron los documentos seleccionados en texto completo a través de la librería de la Universidad McGill (Montreal QC, Canadá).

El siguiente paso fue la extracción de datos. Se usó Microsoft Excel para crear dos formatos basados en las variables que responderían a la pregunta de investigación: un formato para los registros de ensayos clínicos y otro para los documentos restantes. En este paso se hicieron reuniones regulares para discutir y ajustar los formatos.

Se usó el método descriptivo-analítico sugerido por Arksey, *et al.*^18^, para recolectar la información estándar de cada documento. Primero, se calibró el formato usando el 5 % de los documentos para determinar si el método de extracción de datos era adecuado. A continuación, se extrajo la información de todos los documentos restantes.

Se extrajo la siguiente información cuando estaba disponible: autores, título del documento, tipo de documento (comentario, carta al editor, estudio *in vitro,* estudio clínico), objetivo, idioma, fecha de publicación, revista, país de los autores, y hallazgos principales. En el caso de los registros de ensayos clínicos, se recolectó la siguiente información: código de identificación, estado de reclutamiento, diseño del ensayo, país, tamaño de muestra, intervención, control, medida primaria de resultado, fecha de inicio o de registro, fecha anticipada de terminación y fuente.

### Síntesis y presentación de los resultados

Los resultados de la revisión se presentan siguiendo las categorías propuestas por Grudniewicz, *et al.*^22^:

- un resumen de las características y la distribución de las publicaciones incluidas, y
- una síntesis narrativa de los resultados.

En este artículo se empleó la extensión de la declaración PRISMA para reportar revisiones sistemáticas exploratorias (PRISMA-ScR) ^23^. La lista de chequeo diligenciada está disponible en el archivo suplementario 2. El presente estudio no requirió aprobación ética.

## Resultados

**Figura 1:**
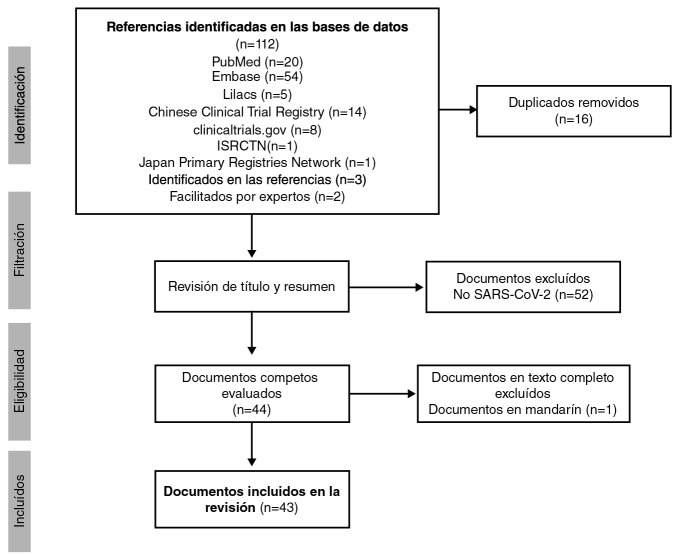
Diagrama PRISMA de la revisión

Se incluyeron 19 documentos y 24 registros de ensayos clínicos (n=43) (figura 1 y archivo suplementario 3).

### Ensayos clínicos de exploración de la efectividad y la seguridad de la cloroquina

Se encontró que el 66 % (16/24) de los ensayos clínicos están registrados en China, dos en el Reino Unido y uno en Estados Unidos, México, Noruega, Corea del Sur, Tailandia y Japón cada uno. Sumando todos los ensayos clínicos, la muestra total es de 18.059 participantes. El ensayo más grande se propone explorar el papel profiláctico de la cloroquina en 10.000 profesionales de la salud a cargo de pacientes con COVID-19 en Inglaterra. Las características de estos ensayos clínicos se describen en el cuadro 1.

**Cuadro 1:** Características de los ensayos clínicos que exploran la efectividad y la seguridad de la cloroquina o sus derivados en pacientes con COVID-19

ID	Diseño del ensayo	País	Tamaño de la muestra	Intervención	Control ^†^	Medida primaria de resultado	Fecha de inicio o registro	Fecha esperada de finalización
NCT04303507	Ensayo controlado aleatorizado paralelo doble ciego ^a^	Inglaterra	10.000	Dosis de carga de 10 mg/ kg de cloro quina seguida por 150 mg al día por 3 meses	Placebo	Número de infecciones sintomáticas COVID-19	Mayo, 2020	Mayo, 2022
ISRCTN86534580	Ensayo controlado aleatorizado pragmático basado en la comunidad a	Reino Unido	3.000	200 mg sulfato de hidroxicloroquina dos al día por 7 días más tratamiento usual	Tratamiento usual	Necesidad de hospitalización por COVID-19	12 de marzo, 2020	24 de marzo, 2020
NCT04308668	Ensayo controlado aleatorizado paralelo de múltiples brazos ^b^	Estados Unidos	1.500	800 mg de hidroxicloroquina seguidos por 600 mg tras 6-8 horas, seguidos por 600 mg al día por 6 días	Placebo siguiendo el régimen de intervención	Incidencia de COVID-19 y escala de gravedad de la infección	17 de marzo, 2020	Mayo, 2021
NCT04286503	Ensayo controlado aleatorizado paralelo ^a^	China	520	Fosfato de cloroquina o lopinavir/ritonavir	Tratamiento básico	Fiebre; resolución de la inflamación pulmonar; conversión negativa de SARS-CoV-19 en frotis de garganta	23 de febrero, 2020	28 de febrero, 2021
NCT04315896	Ensayo controlado aleatorizado de dos brazos, doble ciego, paralelo ^a^	Mexico	500	200 mg de hidroxicloroquina cada 12 horas por 10 días	Placebo siguiendo el régimen de intervención	Comprimido de almidón	23 de marzo, 2020	22 de marzo, 2021
ChiCTR2000029868	Ensayo controlado Aleatorizado multicentrico abierto	China	360	Sulfato de hidroxicloroquina oral	Tratamiento Convencional para COVID-19	Test de ácido nucleico viral	15 de febrero, 2020	No disponible
ChiCTR2000029803	Ensayo controlado aleatorizado abierto	China	320	Hidroxicloroquina	Clorhidrato de arbidol	Progresión de sospechoso a infectado por SARS-CoV-2	14 de febrero, 2020	No disponible
ChiCTR2000029559	Ensayo controlado aleatorizado paralelo	China	300	Hidroxicloroquina	Comprimido de almidón	Conversión viral negativa; tiempo de recuperación de células T	04 de febrero, 2020	No disponible
ChiCTR2000029609	Ensayo controlado Aleatorizado multicéntrico abierto	China	205	Brazo 1: fosfato de Cloroquina Brazo 2: lopinavir/ritonavir	Cloroquina más lopinavir/ritonavir	Conversión viral negativa	06 de febrero, 2020	No disponible
ClinicalTrials.gov: NCT04316377 EudraCT Number: 2020-001010-38	Ensayo controlado aleatorizado de dos brazos pragmático paralelo ^a^	Noruega	202	400 mg hidroxicloroquina dos veces al día por 7 días más tratamiento estándar	Tratamiento estandar	Tasa de disminución en la carga viral de SARSCoV-2	23 de marzo, 2020	03 de marzo de 2025
NCT04307693	Ensayo controlado aleatorizado paralelo multicíntrico b	Corea del Sur	150	400 mg de Hidroxicloroquina cada 12 horas por 7-10 días	Sin hidroxicloroquina	Carga viral SARSCoV- 19	11 de marzo, 2020	Mayo de 2020
ChiCTR2000030987	Ensayo controlado aleatorizado paralelo	China	150	Favapivir mas fosfato de cloroquina	Placebo	Mejoría de síntomas respiratorios	20 de marzo, 2020*	No disponible
ChiCTR2000029741	Ensayo controlado Aleatorizado multicéntrico, abierto	China	112	Fosfato de cloroquina	Lopinavir /ritonavir	Duración de la enfermedad; mortalidad por todas las causas; valores del hemograma, marcadores de inflamación, indicadores de coagulación, ácido nucleico viral	11 de febrero, 2020*	No disponible
ChiCTR2000030054	Ensayo controlado aleatorizado abierto	China	100	Brazo 1: fosfato de Cloroquina Brazo 2: sulfato de hidroxicloroquina	Tratamiento recomendado para COVID-19	Tiempo de mejoría clínica	22 de febrero, 2020*	No disponible
ChiCTR2000029992	Ensayo controlado aleatorizado abierto	China	100	Brazo 1: fosfato de cloroquina Brazo 2: sulfato de hidroxicloroquina	Tratamiento recomendado para COVID-19	Tiempo de mejoría clínica; cambios en la carga viral en muestras de vías respiratorias superior e inferior	18 de febrero, 2020*	No disponible
ChiCTR2000029939	Ensayo controlado aleatorizado	China	100	Fosfato de cloroquina mas tratamiento convencional para COVID-19	Tratamiento recomendado para COVID-19	Duración de hospitalización	16 de febrero, 2020*	No disponible
ChiCTR2000029935	Intervención de un solo brazo	China	100	Fosfato de cloroquina mas tratamiento convencional para COVID-19	Sin grupo de control	Duración de hospitalización	16 de febrero, 2020*	No disponible
NCT04303299	Ensayo controlado aleatorizado paralelo de múltiples brazos ^a^	Tailandia	80	Tres brazos usaran cloroquina mas varios antivirales (oseltamivir, darunavir, ritonavir)	Cuarentena convencional	Tiempo de erradicación del SARS-CoV-2	15 de marzo, 2020	30 de noviembre, 2020
ChiCTR2000030718	Ensayo controlado aleatorizado paralelo	China	80	Fosfato de cloroquina	Nada	Tiempo de mejoría clínica	11 de marzo, 2020*	No disponible
ChiCTR2000029988	Ensayo controlado	China	80	Fosfato de cloroquina	Nada	Tiempo de mejoría clínica	18 de febrero, 2020*	No disponible
jRCTs031190227	Intervención multicéntrica no controlada ^a^	Japon	50	Lopinavir, ritonavir e hidroxicloroquina con o sin oseltamivir	No mencionado	Proteína C reactiva antes y después del tratamiento	27 de febrero, 2020	No disponible
NCT04261517	Ensayo controlado aleatorizado paralelo ^b^	China	30	400 mg de hidroxicloroquina al día por 5 días más tratamiento convencional	Tratamiento convencional	Tasa de eliminación viral en esputo, frotis de garganta o secreciones de vía respiratoria inferior; tasa de mortalidad de participantes	06 de febrero, 2020	31 de diciembre, 2020
ChiCTR2000029542	Ensayo controlado no aleatorizado	China	20	Cloroquina	Manejo convencional	Conversión viral negativa; mortalidad por todas las causas a 30 días	03 de febrero, 2020*	No disponible
ChiCTR2000029975	Intervención de un solo brazo	China	10	Fosfato de cloroquina	Sin grupo de control	Tiempo de conversión viral negativa; mortalidad por todas las causas	18 de febrero, 2020*	No disponible

^a^ No iniciado; ^b^ Iniciado; ^†^ Los detalles del manejo convencional no se describen en los registros; ^*^ Fecha de registro

El 83 % (20/24) de los ensayos fue controlado y aleatorizado, en tanto que dos (8 %) son ensayos controlados no aleatorizados y otros dos intervenciones no controladas. Nueve ensayos evalúan la cloroquina únicamente, ocho exclusivamente la hidroxicloroquina y el resto añade un segundo medicamento a cualquiera de estos dos.

En el caso del grupo de control, en el 54 % (12/22) de los estudios controlados se emplea el manejo convencional para la COVID-19, tres usan placebo, tres, medicamentos (arbidol o lopinavir-ritonavir), y en el resto de los ensayos esta información no se determinó. Los ensayos usarán diferentes medidas primarias de resultados, como carga y conversión viral, duración de la enfermedad, mortalidad, síntomas respiratorios y fiebre, y marcadores sanguíneos como la proteína C reactiva. Tres de ellos (NCT04308668, NCT04307693 y NCT04261517) ya están reclutando participantes (23 de marzo de 2020), aunque esta información no está disponible en el *Chínese Clínica! Tria! Regístry,* por lo que el número es en realidad mayor.

Dos ensayos (NCT04303299 y ISRCTN86534580) planean terminar en el 2020, tres en el 2021, tres en el 2022, uno en el 2020 y el último en el 2025. En el resto de los ensayos la fecha es incierta.

### Síntesis de los hallazgos de las publicaciones incluidas en la revisión

Nueve de los 19 documentos incluidos son comentarios, cartas o editoriales, tres son estudios *in vitro,* dos son revisiones narrativas sobre la farmacología de la cloroquina y la hidroxicloroquina, dos son guías de práctica clínica, uno es una revisión sistemática y otro, un consenso de expertos (archivo suplementario 3). Solo se encontró un ensayo controlado. Dieciséis documentos están en inglés y los otros tres en mandarín, italiano y holandés cada uno. Los documentos en mandarín y holandés tienen resumen en inglés en concordancia con los criterios de inclusión. Ocho documentos son de China, cinco de Francia, dos de Italia, dos de Estados Unidos, uno de Irán y el otro de Holanda. Los hallazgos principales de cada uno de estos documentos se describen en el cuadro 2.

**Cuadro 2:** Características de las publicaciones incluidas en la revisión

Autores	Tipo de documento	Objetivo	Fecha de publicación (año: 2020)	Revista	País de los autores	Hallazgo principal / contribución
Wang M, Cao R, Zhang L, Yang X, Liu J, Xu M, *et al*.	Carta al editor -Estudio *in vitro* ^a^	Evaluar la eficiencia antiviral de cinco medicamentos aprobados por la FDA y dos antivirales de amplio espectro en el COVID-19	04 de febrero	Cell Research	China	Primer estudio en reportar actividad de la cloroquina *in vitro* frente al SARS-CoV-2.
Colson P, Rolain JM, Raoult D	Comentario- sección *Hot* *Topic* ^a^	NA	15 de febrero	International Journal of Antimicrobial Agents	Francia	Los investigadores, expertos en hidroxicloroquina en infecciones intracelulares, resaltan la importancia del hallazgo de Wang, *et al*. Plantean potencial uso profiláctico y terapéutico del medicamento en COVID-19.
Multicenter collaboration group of Department of Science and Technology of Guangdong Province for chloroquine in the treatment of novel coronavirus pneumonia	Consenso de expertos ^b *^	Guiar y regular el uso de cloroquina en pacientes con neumonía por SARS-CoV-2	20 de febrero	Zhonghua Jie He He Hu Xi Za Zhi (Chinese Journal of Tuberculosis and Respiratory Diseases)	China ^*^	Recomendó el tratamiento de COVID-19 con fosfato de cloroquina, 500 mg, dos veces al día, durante 10 días para pacientes diagnosticados con neumonía por SARS-CoV-2 leve y moderada a grave, sin contraindicación para la cloroquina.
Dong L, Hu S, Gao J	Comentario ^a^	NA	29 de febrero	Drug Discoveries & Therapeutics	China	Comenta que el fosfato de cloroquina ha sido incluido en la sexta edición de las guías para la prevención, diagnóstico, y tratamiento de la neumonía por el nuevo coronavirus de la Comisión Nacional de Salud del gobierno chino. Discute otros medicamentos con efectos potenciales sobre el SARS CoV-2 como arbidol, remdesivir, y favipiravir.
Gao J, Tian Z, Yang X	Comentario ^a^	NA	29 de febrero	BioScience Trends	China	Reporta que los resultados en 100 pacientes indican que el fosfato de cloroquina es superior al tratamiento de control en pacientes con neumonía por SARS-CoV-2.
Song P, Karako T	Editorial ^a^	NA	29 de febrero	BioScience Trends	China	Resalta el papel potencial de la cloroquina en el tratamiento de COVID-19.
Colson P, Rolain JM, Lagier JC, Brouqui P, Raoult D	Comentario - seccion *Hot Topic* a	NA	04 de marzo	International Journal of Antimicrobial Agents	Francia	Resalta hallazgos de Wang, *et al*. y Gao, *et al*., y sugiere que la hidroxicloroquina podria ser también efectiva contra el SARS-CoV-2. Resume estudios sobre la actividad de la cloroquina o la hidroxicloroquina en coronavirus (no SARS-CoV-2).
Touret F, Lamballerie X	Comentario ^a^	NA	05 de marzo	Antiviral Research	Francia	Llama la atención sobre la necesidad de interpretar los hallazgos de Wang, *et al.* y Gao, et al. con precaución.
Yao X, Ye F, Zhang M, Cui C, Huang B, Niu P, *et al*.	Estudio i*n vitro* ^a^	Explorar la actividad farmacológica de la cloroquina y la hidroxicloroquina en celulas Vero infectadas con SARS-CoV-2	09 de marzo	Clinical Infectious Diseases	China	Encontraron que la hidroxicloroquina es más potente que la cloroquina in vitro.
Cortegiani A, Ingoglia G, Ippolito M, Giarratano A, Einav S	Revisión sistemática ^a^	Explorar la evidencia sobre la efectividad y la seguridad de la cloroquina para el tratamiento de COVID-19	10 de marzo	Journal of Critical Care	Italia e Israel	Encontraron 6 publicaciones y 23 ensayos clínicos en curso.
Devaux CA, Rolain JM, Colson P, Raoult	Revisión narrativa ^a^	Describir los posibles mecanismos de acción de la cloroquina sobre la replicación de SARS-CoV-2	12 de marzo	International Journal of Antimicrobial Agents	Francia	Describe en detalle los varios mecanismos de acción antivirales de la cloroquina.
Sahraei Z, Shabani M, Shokouhi S, Saffaei A	Carta al editor ^a^	NA	17 de marzo	International Journal of Antimicrobial Agents	Iran	Comenta que en algunos países como Iran, la disponibilidad de la cloroquina es limitada, por lo cual la hidroxicloroquina es una opción valida.
Liu J, Cao R, Xu M, Wang X, Zhang H, Hu H, *et al*.	Carta al editor -Estudio *in vitro* ^a^	Evaluar el efecto antiviral de la hidroxicloroquina contra el SARS-CoV-2, en comparación con la cloroquina in vitro	18 de marzo	Cell Discovery	China	Reportan un efecto más potente de la cloroquina, en comparación con la hidroxicloroquina. Reportan 7 ensayos clínicos en curso que exploran la efectividad de la hidroxicloroquina en China.
Zhou D, Dai SM, Tong Q	Revisión narrativa ^a^	NA	20 de marzo	Journal of Antimicrobial Chemotherapy	China	Resalta el potencial rol de la hidroxicloroquina en modular la "tormenta citoquimica". Comenta que es más segura que la cloroquina y que puede ser usada en mujeres embarazadas. También, que es más económica y está disponible en China.
Gautret P, Lagier J, Parola P, Hoang VT, Meddeb L, Mailhe M	Ensayo controlado ^a^	Evaluar el efecto de la hidroxicloroquina en pacientes infectados con SARS-CoV-2	20 de marzo	International Journal of Antimicrobial Agents	Francia	Primer estudio experimental sobre efectividad de la hidroxicloroquina en pacientes con COVID-19
Sociedad Italiana de Enfermedades Infecciosas y Tropicales (Simit, acrónimo en italiano)	Guía de practica clinica ^c^	NA	Marzo	NA	Italia	Recomienda uso de cloroquina o hidroxicloroquina para manejo de pacientes con COVID-19.
Grupo de Trabajo Holandes sobre Política Antibiótica (SWAB, acrónimo en holandes)	Guía de practica clínica ^d *^	NA	Marzo	NA	Holanda *	Recomienda el uso de cloroquina o hidroxicloroquina para el manejo de pacientes con COVID-19.
Wilson FP	Comentario ^a †^	Revisar la calidad del estudio de Gautret, *et al*.	Marzo, 2026	NA	Estados Unidos	Presenta los fallos metodológicos del estudio.
Hinton DM	Carta ^†^	Autorizar uso de emergencia de medicamentos en Estados Unidos	Marzo, 2028	NA	Estados Unidos	Emitió una Autorización de Uso de Emergencia para el fosfato de cloroquina y el sulfato de hidroxicloroquina en Estados Unidos.

^a^ Inglés; ^b^ Mandarín; ^c^ Italiano; ^d^ Holandés

* Solo se revisaron los resúmenes de estos documentos

† Documentos sugeridos por expertos aparecidos después de marzo 25, 2020

NA: no aplica

El 4 de febrero de 2020, Wang, *et al.,* publicaron en la revista *Cell Research* el primer estudio sobre la actividad de la cloroquina en el SARS-CoV-2 ^11^. Reportaron que esta previene la replicación *in vitro* del virus en células Vero E6 y animaron a la comunidad científica a investigar la efectividad del medicamento en pacientes con COVID-19.

En una publicación del 19 de febrero de 2020, Gao, *et al.,* reportaron que los "resultados de más de 100 pacientes demostraron que el fosfato de cloroquina es superior al tratamiento de control al inhibir la exacerbación de la neumonía, mejorar los hallazgos en las imágenes diagnósticas pulmonares (tomografía axial computarizada), promover la conversión negativa del virus, y acortar la duración de la enfermedad." ^24^. No se reportaron reacciones adversas graves al medicamento.

En su comentario publicado el 5 de marzo de 2020, Touret, *et al.,* piden mesura frente a los hallazgos de Gao, *et al.*^16^. Comentan que si bien el hallazgo es una noticia excelente dado el bajo costo y la disponibilidad de la cloroquina en todo el mundo, los resultados deben interpretarse con precaución, pues los datos que respaldan las conclusiones del estudio aún no están disponibles. Además, según ellos, el estudio de Gao, *et al.,* se realizó en 10 hospitales diferentes, con protocolos clínicos distintos, diversos grupos de control (placebo, otros antivirales, nada, etc.), y variables heterogéneas.

Posteriormente, el 9 de marzo, Yao, *et al.,* publicaron un estudio *in vitro* comparando la actividad farmacológica de la cloroquina y la hidroxicloroquina en células Vero 6 infectadas con SARS-CoV-2. Encontraron que la hidroxicloroquina (EC_50_=0,72 µM) también es eficaz contra el SARS-CoV-2 y es más potente que la cloroquina (EC_50_=5,47 µM) *in vitro*^25^.

El 18 de marzo de 2020, Liu, *et al.,* publicaron un estudio en el que exploraron el efecto antiviral *in vitro* de la hidroxicloroquina contra el SARS-CoV-2 en comparación con la cloroquina ^26^. Reportaron valores de EC_50_ de 2,71, 3,81, 7,14 y 7,36 µM de cloroquina y de 4,51, 4,06, 17,31 y 12,96 µM de hidroxicloroquina para diferentes multiplicidades de infección. Estos datos sugieren un efecto más potente de la cloroquina que de la hidroxicloroquina (diferencias estadísticamente significativas). Usando técnicas de inmunofluorescencia, los autores también reportaron que ambos medicamentos bloquean el transporte del SARS-CoV-2 desde los endosomas tempranos hasta los endolisosomas, lo que parece ser un requisito para liberar el genoma vírico a nivel intracelular. Asimismo, observaron cambios morfológicos de los endosomas tempranos en las células expuestas a cualquiera de los dos medicamentos, lo que sugiere un bloqueo de la maduración en las etapas intermedias de la endocitosis que dificultaría el transporte del virión al sitio de liberación intracelular.

El 20 de marzo de 2020, los investigadores franceses Gautret, *et al.,* publicaron el primer ensayo clínico sobre la efectividad de la hidroxicloroquina en pacientes infectados con SARS-CoV-2 ^27^. El ensayo no fue aleatorizado, pero sí contó con grupo de control; 26 pacientes recibieron 200 mg del medicamento tres veces al día durante 10 días. Algunos de los pacientes recibieron azitromicina adicional para prevenir la infección bacteriana. Después de seis días de tratamiento encontraron, mediante reacción en cadena de la polimerasa en cultivo de frotis nasofaríngeo, que el 70 % de los pacientes en el grupo de intervención estaba virológicamente curado, comparado con el 12,5 % de los 16 pacientes del grupo de control. Más aún, al día seis de la intervención todos los pacientes que habían recibido una combinación de hidroxicloroquina y azitromicina estaban virológicamente curados, en comparación con el 57 % de los pacientes que habían recibido únicamente hidroxicloroquina y del 12,5 % de los pacientes del grupo control. Seis pacientes del grupo de intervención no finalizaron el estudio por diferentes razones: tres casos fueron transferidos a la unidad de cuidados intensivos, uno murió y los otros dos interrumpieron su tratamiento. Estos pacientes no fueron incluidos en el análisis final.

Los autores concluyeron que el tratamiento con hidroxicloroquina se asociaba significativamente con una reducción o desaparición de la carga viral en pacientes con COVID-19 y que, además, este efecto era sinérgico con la azitromicina. Los investigadores recomendaron que los pacientes con COVID-19 sean tratados con hidroxicloroquina y azitromicina para curar su infección y para limitar la transmisión del virus a otras personas. Sugirieron que deben hacerse nuevos estudios para determinar si la hidroxicloroquina puede usarse como quimioprofilaxis contra la COVID-19, especialmente en trabajadores de la salud.

Al analizar el estudio de Gautret, *et al.,* Wilson comentó que hubo pérdida diferencial e importante durante el seguimiento a los pacientes en los dos grupos del estudio ^28^. A diferencia del grupo de intervención, ningún paciente del grupo control murió o fue remitido a la unidad de cuidados intensivos. Según Wilson, si estos seis pacientes hubieran sido incluidos en el análisis, la hidroxicloroquina hubiese estado asociada con un aumento del riesgo de muerte y transferencia a la unidad de cuidados intensivos en los pacientes con COVID-19. El descartar a los seis pacientes mencionados permitió a los autores reportar un aparente impacto positivo de la hidroxicloroquina en su estudio.

El 28 de marzo de 2020, la *Food and Drug Administration* (FDA) de Estados Unidos emitió una "autorización de uso de emergencia" para el fosfato de cloroquina y el sulfato de hidroxicloroquina. La institución autorizó el uso de estos medicamentos para tratar pacientes adultos o adolescentes con peso de 50 kg o más hospitalizados con COVID-19, para quienes la participación en ensayos clínicos no estuviera disponible o no fuera posible ^29^.

## Discusión

Se encontraron 24 registros de ensayos clínicos y 19 documentos sobre el efecto de la cloroquina o la hidroxicloroquina en la COVID-19. La mayoría de los ensayos clínicos son aleatorizados, se desarrollan en China y en total acumulan un tamaño de muestra de más de 18.000 pacientes. Un número similar de ensayos evalúan los efectos de la cloroquina y la hidroxicloroquina, a veces combinadas con un segundo medicamento. La mayoría de los ensayos utiliza el tratamiento convencional en el grupo de control, aunque los registros no describen los componentes del tratamiento convencional y emplean diferentes tipos de variables.

La mayoría de los documentos incluidos son teóricos y solo cuatro son estudios empíricos. De estos, se encontró solamente un ensayo controlado no aleatorizado en humanos. Los datos de los estudios empíricos sugieren que la cloroquina y la hidroxicloroquina son efectivas para controlar la replicación del virus SARS-CoV-2 y, tal vez, para mejorar el curso de la COVID-19. Sin embargo, las conclusiones del único ensayo clínico disponible no son concluyentes ^27^ debido al reducido tamaño de la muestra y al deficiente diseño del estudio ^28^. Tampoco se cuenta con los datos del ensayo clínico de Gao, *et al.,* en 100 pacientes ^24^.

Los autores de los documentos incluidos en esta revisión comentan que la cloroquina y la hidroxicloroquina son medicamentos generalmente seguros, y que los efectos adversos comunes son menores y transitorios ^16^. Sin embargo, dado que especialmente la cloroquina puede ser cardiotóxica y neurotóxica, estos medicamentos deben ser usados con precaución y debe evitarse la automedicación ^16^.

En todo el mundo los investigadores están estudiando la actividad de otros medicamentos contra el SARS-CoV-2, como el lopinavir, el ritonavir, la ribavirina, el penciclovir, la nitazoxanida y el nafamostat. Sin embargo, a diferencia de la cloroquina, dichos fármacos no están ampliamente disponibles para el tratamiento de un número elevado de pacientes con COVID-19. Por lo tanto, de todos estos medicamentos la cloroquina parece el medicamento de mayor potencial para el uso a gran escala debido a su disponibilidad, seguridad y bajo costo ^26^. La FDA ya emitió una "autorización de uso de emergencia" para el fosfato de cloroquina y el sulfato de hidroxicloroquina ^29^, la primera autorización de este tipo emitida en Estados Unidos. Compañías como Sandoz y Novartis donaron 30 millones de dosis para la lucha contra la COVID-19. Además, otras compañías han redoblado la producción para cubrir la creciente demanda en Estados Unidos y otros países ^30^.

### Limitaciones

Debido al poco tiempo de preparación y la urgencia de los resultados, la estrategia de búsqueda de la presente revisión no contó con la orientación de un bibliotecólogo. Sin embargo, nuestro equipo tiene experiencia en la elaboración de revisiones sistemáticas ^31^^-^^36^. Solo se incluyeron tres bases de datos, pero cada una de ellas aporta un aspecto clave a la búsqueda: Embase está especializada en farmacología, PubMed es la base de datos médica más nutrida del mundo, y Lilacs es la base de datos latinoamericana más completa. No incluimos documentos exclusivamente en mandarín, pero incluimos estudios en cinco idiomas.

La revisión no incluyó una evaluación de la calidad de los datos, pues las revisiones sistemáticas exploratorias, por lo general, no incluyen este tipo de evaluaciones, ya que a diferencia de las revisiones sistemáticas clásicas, la pregunta de investigación es menos específica ^18^. Además, este tipo de estudios reporta la evidencia independientemente de su calidad ^37^. La extensión de la declaración PRISMA para el reporte de revisiones sistemáticas exploratorias ^23^, la cual se siguió en este estudio, desaconseja realizar análisis de la calidad de la información. Por último, los hallazgos que se presentan reflejan la escasa información disponible y el deficiente diseño de los estudios encontrados.

## Conclusiones

La utilización de medicamentos conocidos para enfrentar la COVID-19 es una estrategia práctica porque ya se cuenta con información sobre los efectos adversos, la posología y las interacciones farmacológicas. A la luz de la información disponible, se recomienda el uso de la cloroquina y la hidroxicloroquina para el tratamiento de pacientes con COVID-19 única y exclusivamente bajo autorización y vigilancia médica en ensayos controlados aleatorizados. El uso de estos medicamentos por fuera del ámbito clínico puede ocasionar reacciones adversas potencialmente fatales, por lo que su venta sin vigilancia y su uso indiscriminado deben controlarse estrictamente.

Hasta el momento, solo un ensayo clínico no aleatorizado con un reducido tamaño de muestra e importantes deficiencias, ha reportado que la hidroxicloroquina es efectiva para eliminar el SARS-CoV-2 en seres humanos. Es urgente el acceso a análisis interinos de otros ensayos clínicos para determinar la efectividad y la seguridad de la cloroquina y la hidroxicloroquina en pacientes con COVID-19. Se sabe de por lo menos dos ensayos clínicos cuyos resultados están previstos para el 2020.

Además de averiguar el perfil de seguridad y la efectividad de los medicamentos para controlar la COVID-19, es importante saber si es posible recomendar la cloroquina o la hidroxicloroquina como quimioprofilaxis para los profesionales de la salud que se infectan en sus labores y mueren por esta enfermedad. También debe determinarse si el efecto clínico depende de la edad de los pacientes, la presentación clínica o la etapa de la enfermedad. Estas son preguntas clave que deberán ser respondidas en futuros estudios clínicos.

## Archivo suplementario 1

1. Estrategia de búsqueda (última actualización, marzo 25 de 2020)

**Table:**

Pubmed
((((((Coronavirus[Title]) OR COVID[Title]) OR SARS-CoV-2[Title/Abstract]) OR “severe acute respiratory syndrome coronavirus 2” [Supplementary Concept]) OR “COVID-19” [Supplementary Concept])) AND (((“Aminoquinolines”[Mesh]) OR chloroquine[Title]) OR hydroxychloroquine[Title])
Embase
1. exp SARS coronavirus/ or exp Coronavirinae/
2. exp aminoquinoline derivative/
3. exp chloroquine/
4. hydroxychloroquine/
5. 2 or 3 or 4
6. 1 and 5
Lilacs
(“Covid” OR “Coronavirus” OR “SARS-CoV-2” OR “Virus del SARS”) AND (Aminoquinolinas OR Cloroquina OR Hidroxicloroquina)

2. Número de ensayos clínicos incluidos en cada base de datos (búsqueda, marzo 23, 2020)

**Table:**

Nombre	Sitio Web	Incluidos
U.S. National Library of Medicine	clinicaltrials.gov	8
Australian New Zealand Clinical Trials Registry (ANZCTR)	anzctr.org.au	0
Brazilian Clinical Trials Registry (ReBec)	ensaiosclinicos.gov.br	0
Chinese Clinical Trial Registry	chictr.org.cn	14
Clinical Research Information Service (CRiS), Republic of Korea	cris.nih.go.kr	0
Clinical Trials Registry - India (CTRI)	ctri.nic.in/Clinicaltrials/advsearch.php	0
Cuban Public Registry of Clinical Trials(RPCEC)	registroclinico.sld.cu/en/home	0
EU Clinical Trials Register (EU-CTR)	clinicaltrialsregister.eu	0
German Clinical Trials Register (DRKS)	drks.de/drks_web/	0
Iranian Registry of Clinical Trials (IRCT)	irct.ir	0
ISRCTN	isrctn.com/	1
Japan Primary Registries Network (JPRN)	rctportal.niph.go.jp/	1
The Netherlands National Trial Register (NTR)	trialregister.nl/	0
Pan African Clinical Trial Registry (PACTR)	pactr.samrc.ac.za/	0
Peruvian Clinical Trial Registry (REPEC)	ensayosclinicos-repec.ins.gob.pe/	0

Los términos de búsqueda en cada base de datos fueron: (Aminoquinolinas OR Cloroquina OR Hidroxicloroquina)

### Archivo suplementario 2

PRISMA Extension for Scoping reviews (PRISMA-ScR) 2018 Checklist^1^

**Table:**

Section/topic	#	PRISMA-ScR Checklist item	Reported on page #
TITLE			
Title	1	Identify the report as a scoping review.	1
ABSTRACT			
Structured summary	2	Provide a structured summary including, as applicable: background; objectives; data sources; study eligibility criteria, participants, and interventions; study synthesis methods; results; limitations; conclusions and implications of key findings.	1
INTRODUCTION			
Rationale	3	Describe the rationale for the review in the context of what is already known. Explain why the review question(s)/objective(s) lend themselves to a scoping review approach.	2-3
Objectives	4	Provide an explicit statement of the question(s) and objective(s) being addressed with reference to their key elements (e.g., population or participants, concepts and context), or other relevant key elements used to conceptualize the review question(s) and/or objective(s)).	3
METHODS			
Protocol and registration	5	Indicate if a review protocol exists, if and where it can be accessed (e.g., Web address), and, if available, provide registration information including registration number.	3
Eligibility criteria	6	Specify the characteristics of the sources of evidence (e.g., years considered, language, publication status) used as criteria for eligibility, and provide a rationale.	3
Information sources	7	Describe all information sources (e.g., databases with dates of coverage, contact with study authors to identify additional sources) in the search and date last searched.	3-4
Search	8	Present full electronic search strategy for at least one database, including any limits used, such that it could be repeated.	Archivo suplementario 1
Selection of sources of evidence	9	State the process for selecting studies (i.e., screening, eligibility) included in the scoping review. Describe the methods of charting data from the included sources of evidence (e.g. piloted forms; forms that	4
Data charting process	10	have been tested by the team before their use, whether data charting was done independently, in duplicate) and any processes for obtaining and confirming data from investigators.	4
Data items	11	List and define all variables for which data were sought and any assumptions and simplifications made.	4
Critical appraisal of individual sources of evidence	12	If done, provide a rationale for conducting a critical appraisal of included sources of evidence; describe the methods used and how this information was used in any data synthesis (if appropriate).	NA
Summary measures	13	Not applicable for scoping reviews.	NA
Synthesis of results	14	Describe the methods of handling and summarizing the data that were charted.	4
Risk of bias across studies	15	Not applicable for scoping reviews.	NA
Additional analyses	16	Not applicable for scoping reviews.	NA
RESULTS			
Selection of sources of evidence	17	Give numbers of studies screened, assessed for eligibility, and included in the review, with reasons for exclusions at each stage, ideally using a flow diagram.	Figura 1
Characteristics of sources of evidence	18	For each source of evidence, present characteristics for which data were charted and provide the citations.	Tablas 1 y 2
Critical appraisal within sources of evidence	19	If done, present data on critical appraisal of included sources of evidence (see item 12).	NA
Results of individual sources of evidence	20	For each included source of evidence, present the relevant data that were charted that relate to the review question(s) and objective(s).	Tablas 1 y 2
Synthesis of results	21	Summarize and/or present the charting results as they relate to the review question(s) and objective(s).	5-10
Risk of bias across studies	22	Not applicable for scoping reviews.	NA
Additional analysis	23	Not applicable for scoping reviews.	NA
DISCUSSION			
Summary of evidence	24	Summarize the main results (including an overview of concepts, themes, and types of evidence available), explain how they relate to the review question(s) and objectives, and consider the relevance to key groups	10-11
Limitations	25	Discuss the limitations of the scoping review process.	11
Conclusions	26	Provide a general interpretation of the results with respect to the review question(s) and objective(s), as well as potential implications and/or next steps.	12
FUNDING			
Funding	27	Describe sources of funding for the included sources of evidence, as well as sources of funding for the scoping review. Describe the role of the funders of the scoping review.	1
